# Effect of in-office bleaching on surface roughness of additively versus subtractively manufactured tooth-colored materials used for fixed definitive prostheses

**DOI:** 10.1186/s12903-025-07454-5

**Published:** 2026-02-02

**Authors:** Zahraa Hassan Ali Sharshar, Waleed Elshahawy, Dina Mohamed Elshokafy

**Affiliations:** 1 Fixed Prosthodontics Department, Faculty of Dentistry, Tanta University, Tanta, Egypt; 2 Mandara, Alexandria, Egypt

**Keywords:** In-office bleaching, Surface roughness, Additively, Subtractively

## Abstract

**Background:**

In-office dental bleaching is recognized as a conservative and effective approach for achieving tooth whitening. Although previous studies have investigated additively manufactured composite resins, there is limited knowledge in terms of the impact of in-office bleaching on the surface roughness of these materials. The goal of this study was to determine the impact of in-office bleaching on the surface roughness of tooth-colored materials used for definitive fixed prostheses, which were fabricated via two different techniques: subtractive and additive manufacturing.

**Methods:**

Two additively manufactured resins, Varseosmile crown plus (VS) and Crowntec (CT), and one subtractively manufactured polymer-infiltrated ceramic, Vita enamic (EN), were applied to fabricate rectangular-shaped specimens (14 × 12 × 1 mm) (*n* = 20). The surface roughness of each sample was measured via a noncontact profilometer before the in-office bleaching material (control) was processed. The bleaching agent (BMS White, Pisa, Italy) was then applied in three consecutive sessions of 15 min each (total 45 min), after which surface roughness was measured again via the same profilometer. For the purpose of statistical evaluation, one-way ANOVA and paired-samples t tests were performed. A *p* value < 0.05 was considered statistically significant, whereas a p-value < 0.001 was considered highly statistically significant.

**Results:**

The mean surface roughness [µm] prior to exposure to the bleaching material did not differ markedly among the groups. Following application of the bleaching agent, both the VS and CT groups demonstrated a notable increase in surface roughness (*P* < 0.001). In contrast, the EN group demonstrated a nonsignificant change in surface roughness.

**Conclusions:**

The tested additively manufactured resins had greater surface roughness than did the subtractively manufactured polymer-infiltrated ceramic, which was more resistant to roughening after all the in-office bleaching procedures were completed.

## Background

Digital technology has significantly transformed various facets of daily life, including the discipline of dental science. Within the discipline of clinical dentistry, computer-aided design/computer-aided manufacturing (CAD/CAM) technologies are utilized and can be stratified into two primary methods: the subtractive manufacturing method and the additive manufacturing method [1].

The additive manufacturing approach, commonly referred to as 3D printing, involves fabricating objects by sequentially layering materials according to 3D model data. The use of 3D printers is rapidly increasing within digital dentistry. This technology has addressed the limitations of conventional dental systems through advancements in both material development and printer capabilities [2].

Regarding the additive manufacturing of polymer-based restorative materials, composite resins designed for definitive fixed prostheses represent some of the most recent advancements in restorative dental materials [3].

The additively manufactured tooth-colored definitive dental material Varseosmile Crown Plus (VS) is now commercially available. It is authorized for individual tooth applications, including full crowns, inlays, onlays, and veneers. This substance is classified as a hybrid composite comprising a methacrylic ester matrix reinforced with ceramic fillers and is categorized as a resin matrix ceramic [4]. Subsequently, additional materials, including Crowntec (CT), were introduced. Manufacturers classify this material as a composite resin, suitable for both tooth-supported and implant-supported restorations [5]. This material has demonstrated performance comparable to that of certain CAD/CAM, bulk-fill, and standard resin composites, suggesting its potential suitability for prolonged restorative applications [6].

The importance of dental aesthetics is widely recognized, contributing to a growing number of patients seeking treatment to enhance their appearance. A frequent goal among these individuals is the attainment of whiter, more radiant teeth [7].

Bleaching is a technique applicable to both natural and restored teeth and aims to achieve a shade closer to the desired or standard level of whiteness [8]. One effective way to eliminate tooth discoloration is to use hydrogen peroxide (HP) for in-office bleaching. This method employs a high-concentration HP solution (35–40%), which oxidizes stains and pigments present over the dental substrate [9]. The primary benefit of in-office bleaching lies in its capacity to whiten teeth within a single dental appointment. However, this procedure may also result in tooth sensitivity and soft tissue irritation [10].

During the bleaching procedure, bleaching agents interact not only with dental surfaces but also with the surfaces of existing dental restorations [11]. While bleaching procedures are considered biocompatible and efficacious for hard dental tissues, they may compromise restorative materials, leading to potential surface deterioration, alterations in surface roughness, erosion, and eventually failure of restoration [12].

Surface roughness is a key factor influencing the longevity of a prosthesis, as increased roughness can promote plaque deposition and biofilm formation, in addition to causing aesthetic issues such as discoloration [13].

Although additively manufactured composite resins are recommended for definitive prostheses, the principal gap in current knowledge involves their microstructural behavior under bleaching conditions compared with milled CAD/CAM materials, and the clinical implications of these surface alterations [13, 14].

Accordingly, two null hypotheses were formulated. The first null hypothesis states that “In-office bleaching does not influence the surface topography of additively and subtractively manufactured tooth-colored materials used for fixed definitive prostheses.” The second null hypothesis assumes that “the category of the material used for fixed definitive prostheses does not influence surface roughness following in-office bleaching.”

## Methods

### Sample size calculation

The estimated number of specimens needed for this study, accounting for the anticipated withdrawal rate, was determined to be 60 specimens. The sample selection was guided by data from a previous study [13]. The threshold for statistical significance in this study was established at 0.05, with a target sample power exceeding 80%, a confidence interval of 95%, and an observed power of 96.77%. The estimated number of samples was ascertained via G\*Power 3.1.9 software. The materials utilized in this research are presented in Table 1.

**Table 1:** Materials tested in this study

Material	Manufacturer	Product	Composition
Additively manufactured hybrid resin	Bego, Bremen, Germany	Varseosmile crown plus	Esterification products of 4.4-isopropylphenol, ethoxylated and 2-methylprop-2enoic acid, silanized dental glass, methyl benzoylformate, diphenyl (2,4,6-trimethylbenzoyl) phosphine oxide. Total content of inorganic filles 30-50 wt%
Additively manufactured hybrid resin	Saremco Dental AG, Rebstein, Switzerland	Crown tec	Esterification products of 4.4-isopropylphenol, ethoxylated and 2-methylprop-2enoic acid, silanized dental glass, pyrogenic silica, initiators. Total content of inorganic fillers: 30-50 wt%
Subtractively manufactured polymer infiltrated ceramic	Vita Zahnfabrik, Bad Sackingen, Germany	Vita enamic	14 wt % methacrylate polymer (urethane dimethacrylate and triethylene glycol dimethacrylate). 86 wt % fine-structure feldspar ceramic network

This invitro study was executed on 60 specimens; the samples were evenly divided into three groups. Two additively manufactured resins, Varseosmile crown plus (VS) (Bego, Bremen, Germany) and Crowntec (CT) (Saremco Dental AG, Rebstein, Switzerland) with standardized A3 shade, along with a subtractively manufactured polymer-infiltrated ceramic network material, Vita enamic (EN) (VITA Zahnfabrik, Bad Säckingen, Germany) in shade 3M2-T.

### Specimen preparation

For the fabrication of the subtractively manufactured samples, rectangular samples measuring (14 mm in length × 12 mm in width × 1 mm in thickness) were sectioned from CAD/CAM blocks of EN material via an automatic slow-speed diamond cutting saw (Isomet 4000, Buehler, Lake Bluff, Illinois, USA) via a water-based cooling system at a cutting speed of 2500 rpm.

For the fabrication of additively manufactured samples, a rectangular-shaped standard tessellation language (STL) file with the same dimensions of 14 × 12 × 1 mm was designed in CorelDRAW X8 software for accurate dimensional layout of the specimens and imported to a digital light processing (DLP) assisted 3D printer (Shining 3D Tech. Co., Ltd., Hangzhou, China) to produce 20 samples from each material. All specimens were printed with a layer thickness of 50 μm, a build orientation of 0° (horizontally aligned to the build platform), and a printing resolution of 75 μm (XY) according to manufacturer recommendations.

After fabrication, the VS samples were pretreated with an ultrasonic cleaning bath containing a durable ethanol solution (95% ethanol Absolut; Grogg Chemie AG), followed by ultrasonic cleaning in fresh 95% ethanol in accordance with the manufacturer’s guidelines, whereas the CT samples were wiped with a cloth saturated with 96% alcohol to eliminate residual resin. Each sample was air-dried with a syringe immediately after cleaning [15].

Subsequent to the cleaning of the specimens, they were light-polymerized with a xenon curing device (Dentacolor XS, Heraus Kulzer, Germany) according to manufacturer’s instructions as follows. The VS samples were light-polymerized 180 s, with 90 s on each side of each sample, and the CT samples were light polymerized 360 s, 180 s on each side.

### Polishing of the specimens

All the specimens were polished in the following order:

A-EVE Diacomp Plus Twist polishing system (EVE Ernst Vetter GmbH, Keltern, Germany) was applied in two consecutive stages, operating at 3000–8000 rpm (not exceeding 10,000 rpm), for 15 s per step [16–18]: starting with medium-grit spiral-shaped pink rubber polishing discs designed for preliminary polishing, followed by fine-spiral-shaped gray rubber polishing discs for final high-gloss polishing.

B- Goat-hair brush (ENA SHINY S brush wheel, Italy) and felt polishing wheel (ENA SHINY F, Italy): Diamond polishing paste **(**Direct Dia Paste, Shofu Dental, Ratingen, Germany) was applied to the specimen surfaces, followed by polishing with a goat hair brush and subsequently a felt wheel, each operated at 10,000 rpm for 15 s [19].

All polishing procedures were performed on a single surface of each specimen and adjusted to simulate clinical intraoral conditions. Polishing was performed in unidirectional motion, with the instrument held perpendicular to the sample surface, following the manufacturer’s recommended speed and duration, and uniform gentle manual pressure was consistently applied by a single operator [20]. Following hand-executed polishing, the samples were subjected to ultrasonic cleaning for 10 min and subsequently air-dried [21]. The final dimensions of each sample were confirmed after finishing using a digital caliper for standardization.

### Control measurements

Following the polishing procedures, baseline surface roughness measurements for each specimen were obtained via a noncontact profilometer. Three distinct areas on the central region of each specimen, one located centrally and two located 2 mm away from the center, were evaluated to assess the surface roughness values, which were then calculated as the mean to obtain the average surface roughness for each specimen. 

### Bleaching of the specimens

 The samples were subjected to 38% HP gel (BMS White Pisa, Italy) by the application of only one layer of bleaching material to cover the entire top surface of each sample over the polished surface [22]. The samples were then exposed to a LED power bleaching system (COXO C- Bright. Medical Instrument Co. Ltd, Foshan, China) as a source of light activation for 15 min. Then, the existing gel was cleared via wet cotton, and a fresh layer of gel was applied for an additional 15 min. This procedure was repeated once more for a further 15-minute session, resulting in a total application time of 45 min [23]. The light-curing parameters were as follows: input power AC 100–240 V, 50/60 Hz; wavelength range 420–500 nm; maximum light intensity > 1000 mW/cm² and light source consisting of six high-power LEDs.

The treated samples were cleaned with distilled water for one minute after each bleaching cycle ended to remove any remaining bleaching chemicals. When not subjected to bleaching, the samples were preserved in distilled water at 37 °C for 24 h [13].

### Surface roughness measurements

The surface roughness of all specimens was assessed via a noncontact profilometer (NANOVEA Inc, Irvine, United States). At a constant level of magnification of 90X, images of the spesimens were taken with a USB digital microscope equipped with an integrated camera (U500X Capture Digital Microscope; Shenzhen Texon Technology Ltd., Guangdong, China) connected to a compatible desktop computer. A 3D depiction of each sample was obtained and analyzed via WSxM software (version 5 develop 4.1, Nanotec Electronica, SL).

### Statistical analysis

The data obtained were organized and analyzed via SPSS version 25 (Statistical Package for the Social Sciences; IBM, Armonk, NY, USA). Descriptive statistics, inclusing average, standard deviation (SD), and range (minimum–maximum), were calculated. Group comparisons were executed via one-way ANOVA (F) tests, with pairwise comparisons conducted via the least significant difference (LSD) method. The variations in surface roughness observed before and after bleaching within the same material were assessed via paired samples t tests. A p-value of < 0.05 was considered statistically significant, whereas pvalues < 0.001 were highly significant.

## Results

The average values and standard deviations of surface roughness prior to exposure to the in-office bleaching material for all groups are shown in Table 2. The findings revealed no notable differences among the groups (*P* = 0.158).

**Table 2:** Comparison between groups as regards Average Roughness [µm] before exposure to the bleaching

	Group EN	Group VS	Group CT
Average Roughness [µm] before exposure to the bleaching			
Min. - Max.	0.139 0.353	0.130 0.375	0.184 0.359
Mean ± SD	0.269 ± 0.041	0.256 ± 0.052	0.270 ± 0.033
ANOVA test	F=1.867, P=0.158		

Following contact with the bleaching agent, the mean surface roughness values increased. This increase was significantly greater in the VS and CT groups, whereas it was not significant in the EN group. No marked difference was documented between the VS and CT groups. These values are detailed in Table 3, with P1 representing the significance of the comparison between the EN and VS groups, P2 presenting the significance of the comparison between EN and CT, and P3 presenting the significance of the comparison between VS and CT.

**Table 3:** Comparison between groups as regards Average Roughness [µm] after exposure to the bleaching

	Group EN	Group VS	Group CT
Average Roughness [µm] after exposure to the bleaching			
Min. - Max.	0.181 ­ 0.360	0.247 ­ 0.496	0.251 ­ 0.468
Mean ± SD	0.271 ± 0.038	0.406 ± 0.059	0.395 ± 0.054
ANOVA test	F= 130.287, P<0.001**		
Post hoc test	P1<0.001**, P2<0.001**, P3=0.219		

**Fig. 1:**
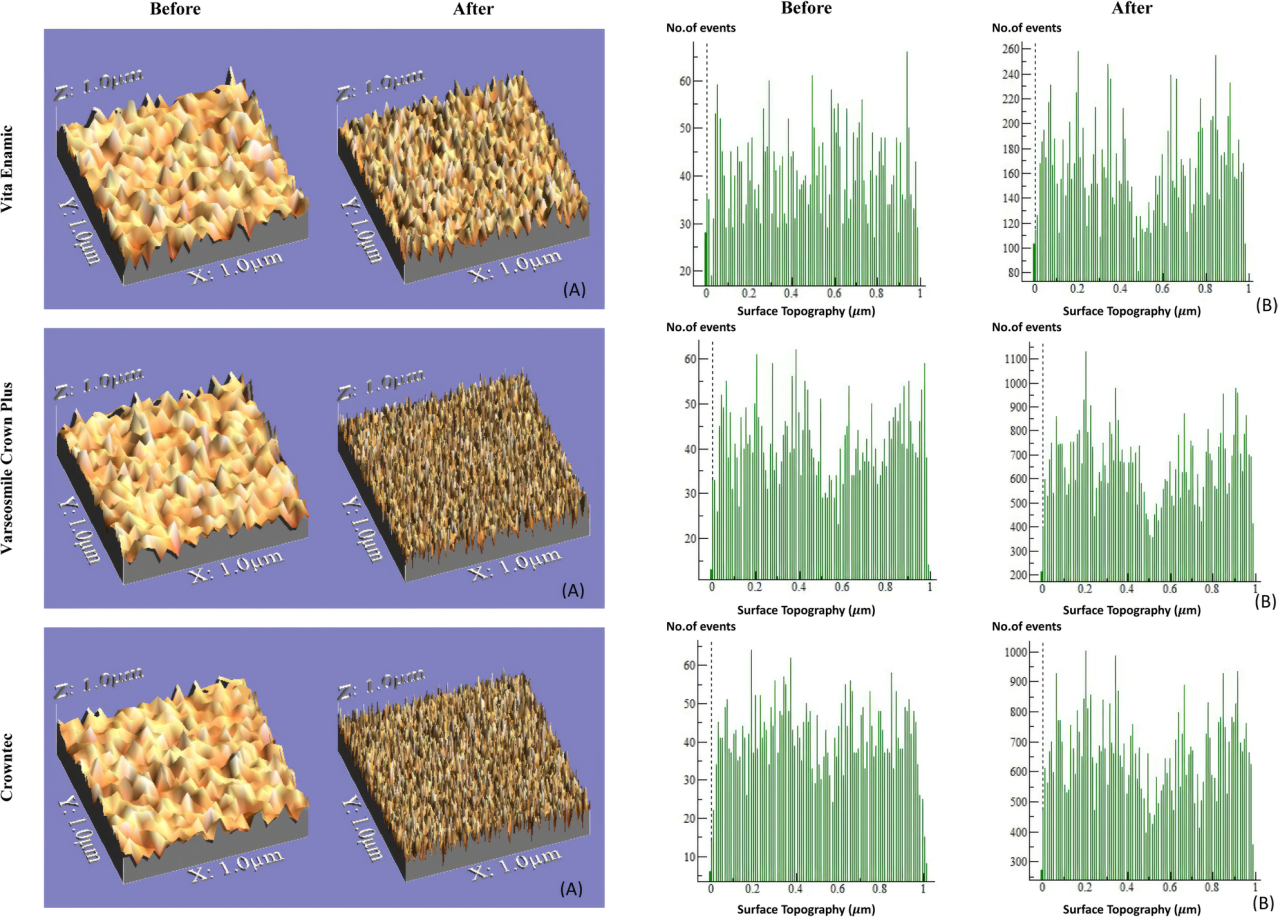
Surface roughness of Vita enamic, Varseosmile crown plus, and Crowntec before and after application of in-office bleaching material. (**A)** 3D images of the surface Topography converted by computer software and (**B)** mathematical histogram

The surface roughness of all the samples was further determined before and after in-office bleaching via microscopic images obtained via a digital microscope. These images were then processed by computer software to generate three-dimensional surface morphologies and corresponding mathematical histograms, as illustrated in Fig. 1.

## Discussion

This research explored the potential effects of in-office bleaching on the surface roughness of additively and subtractively manufactured tooth-colored materials used for fixed definitive prostheses. Guided by the study results, both null hypotheses were rejected: in-office bleaching increased the surface roughness of all evaluated materials, and the type of material used for fixed definitive prostheses influenced the surface roughness following bleaching.

With respect to the selection of materials, a recently introduced type of CAD/CAM dental material, resin-ceramic combination materials, was recently introduced. These materials combine the advantageous attributes of resin composites and ceramics, providing an optimal balance of esthetic morphology and mechanical strength while supporting the least invasive procedures [4]. Accordingly, VS and CT were chosen for inclusion in this study.

According to Mandurino et al., [24]lasting materials for 3D printing remain in the early stages of development, and both in vitro and in vivo studies are essential to define their medical recommended uses, along with those of competing materials. EN was selected for this study because it is clinically established as a polymer-network-based material suitable for the fabrication of definitive prostheses, which aligns with the intended applications of VS and CT materials [16]. Previous reports indicate that the integration of ceramic and polymer stages endows these materials with strengths and durabilities comparable to those of natural teeth [25, 26].

A comparative analysis between the previously mentioned novel materials and traditional CAD-CAM milled materials crucial for assessing their viability and longevity in clinical applications.

A DLP 3D printer was chosen for use in this study. Owing to its advantages over conventional stereolithography printers. DLP technology allows for the simultaneous curing of entire layers, resulting in significantly faster printing speeds. Additionally, it offers high accuracy, excellent surface quality, and cost-effectiveness, making it a suitable choice for efficient and precise fabrication [27].

Since postcuring has a significant effect on the final properties of 3D-printed materials, it is necessary for additively manufactured samples [28]. A non-polymerization apparatus was chosen for this procedure in accordance with the manufacturer’s suggestions. It has the advantage of emitting a broad-spectrum wavelength, which effectively activates a wide range of photoinitiator systems. As a result, it facilitates optimized polymerization, resulting in enhanced conversion and improved mechanical properties [29].

Reymus et al. [30]concluded that the appropriate after-curing strategy for each material is critically important for verifying the optimal mechanical features of 3D-printed resin materials.

To ensure harmonization, all polishing was executed with a single hand operator in the same direction following the manufacturer’s instructions.

According to Alamoush et al., [31] in-office bleaching has been shown to significantly affect the surface roughness of unpolished CAD/CAM composite blocks, indicating that preemptive polishing of restorations prior to bleaching is advisable. Bleaching procedures may modify the structural and physical properties of dental restorative materials, potentially compromising their durability and leading to premature failure [32]. In-office protocols frequently utilize high concentrations of HP, such as 38% H₂O₂, owing to their proven efficacy in achieving rapid whitening results in a clinical setting, BMS white bleaching material was chosen [34].

The thickness of the tested materials was set at 1 mm, representing the minimum recommended thickness for their use in fixed restorations [33]. An electronic caliper was employed to verify the thickness of each sample, thereby minimizing any factors that could influence the ultimate outcomes regarding surface roughness [33].

In our research, all the samples were kept in saline at 37 °C to simulate intraoral fluids and temperature, providing conditions that more closely reflect the clinical environment [33].

Surface roughness is the most widely used method for determining the surface characteristics of various restorative materials. The evaluation of surface roughness helps determine the durability of the restoration by reducing the deposition of dental plaque, discoloration of the restoration, abrasion tendency and damage to periodontal tissue over time [34]. According to Hafez et al. [35], the resin matrix of restorative materials may undergo chemical degradation due to the concentration or repeated administration of peroxide. Furthermore, if the bleaching agent degrades the coupling agent in resin composites, the resulting surface roughness could be exacerbated.

A noncontact profilometer was employed in the present study, as it is considered a preferable and more reliable method. Unlike linear profilometers, it can assess roughness over entire surface areas rather than along limited paths, providing a more precise representation of surface topography. Additionally, it generates high-resolution images of the investigated surfaces and avoids potential surface damage that could introduce bias into the results [36].

With respect to the surface roughness results of our study, the mean value of surface roughness change significantly increased for additively manufactured materials andnonsignificantly increased for subtractively manufactured materials.

The documented increase in surface roughness across all materials may be attributed to the effects of reactive free radicals generated by peroxides at the resin–filler junction, which can induce partial or complete debonding of the filler–matrix interface and promote water uptake, thereby increasing the surface roughness of restorative materials. Additionally, these free radicals and infiltrating water molecules can penetrate the resin matrix and interact with glass particles, silica, and alumina, resulting in the detachment of fillers [37].

The difference in surface roughness between milled hybrid materials and 3D-printed CRC materials can be explained by the more homogeneous filler distribution in the milled materials relative to the 3D-printed materials, which exhibit clusters, stratified macrostructures with spherical pores formed through layer‒by layer polymerization. The relatively low surface roughness of milled hybrid materials relative to 3D-printed CRC materials is likely linked to the intrinsic properties of the material, including a higher fraction of inorganic fillers (86 wt%), a smaller particle size, and a more uniform dispersion of constituents.

This finding is in line with that of Duarte et al., [38] who reported that the filler content of commercially available 3D-printed CRCs is significantly lower than that of clinically established CAD-CAM-machinable CRCs or current direct composite resins.

The data of our study are consistent with those of Yu Hao et al., [12] who investigated the impact of HP bleaching on the physical characteristics of several dental restorative materials and reported that bleaching produced increased surface roughness in all tested restorative materials, except for ceramics. Similarly, Karakaya et al. [39] reported that bleaching did not influence the surface roughness or topography of tested materials, including Lava Ultimate and EN.

Conversely, Karakaya et al. [34] observed that hybrid CAD/CAM blocks were more strongly influenced than composite resins when exposed to high-concentration HP bleaching agents on stained CAD/CAM blocks and a nanohybrid composite resin. However, the exposure of samples to the staining solutions prior to bleaching may have contributed to the variations noted compared with the present study.

Karanasiou et al. [40] assessed the effect of Er, Cr: YSGG laser-assisted tooth bleaching on the surface roughness of PIC and nanocomposite materials and reported that the surface roughness of the tested materials was not notably altered regardless of the bleaching technique.

The study limitations include that the study was executed under laboratory conditions, which fail to completely reproduce the complex and dynamic oral environment. In the oral cavity, factors such as salivary flow, thermal cycling, pH fluctuations, dietary influences, and mechanical wear may alter the surface characteristics and behavior of restorative materials over time. Therefore, further in vivo studies are necessary to evaluate the long-term effects of bleaching on the clinical performance of both 3D-printed and milled restorative materials, particularly in the presence of saliva, dietary stains, and mechanical wear. Additionally, using an ultrasonic cleaning protocol in 95% ethanol consistently to all additively manufactured resin materials, would improve standardization and comparability across specimens.

## Conclusions

Considering the limitations of this in vitro study, the following conclusions are suggested:

1. In-office bleaching increases the surface roughness of both additively and subtractively manufactured materials tested in this study.
2. Additively manufactured materials show greater surface roughness than do subtractively manufactured materials, which demonstrates greater resistance to roughening following in-office bleaching.

Further clinical studies are recommended to closely mimic the circumstances of the oral cavity and to assess the clinical implications of bleaching 3D-printed materials more accurately.

## Data Availability

The datasets used and/or analyzed during theCurrent studies are available from the corresponding author upon reasonable request.

## Abbreviations

STL: Standard Tessellation Language file
CAD/CAM: Computer-aided design/computer-aided manufacturing
HP: Hydrogen peroxide
VS: Varseosmile crown plus
CT: Crowntec
EN: Vita enamic
